# EGFR signaling promotes resistance to CHK1 inhibitor prexasertib in triple negative breast cancer

**DOI:** 10.20517/cdr.2020.73

**Published:** 2020-12-05

**Authors:** Kevin J. Lee, Griffin Wright, Hannah Bryant, Leigh Ann Wiggins, Michele Schuler, Natalie R. Gassman

**Affiliations:** ^1^Department of Physiology and Cell Biology, University of South Alabama College of Medicine, Mobile, AL 36688, USA.; ^2^Mitchell Cancer Institute, University of South Alabama, Mobile, AL 36604, USA.; ^3^Department of Comparative Medicine, University of South Alabama College of Medicine, Mobile, AL 36688, USA.; ^4^Department of Microbiology and Immunology, University of South Alabama College of Medicine, Mobile, AL 36688, USA.

**Keywords:** Triple negative breast cancer, CHK1, replication, apoptosis, drug resistance, epidermal growth factor receptor, mitogenic signaling

## Abstract

**Aim**: Innate resistance to the CHK1 inhibitor prexasertib has been described, but resistance mechanisms are not understood. We aimed to determine the role epidermal growth factor receptor (EGFR) plays in innate resistance to prexasertib in triple negative breast cancer (TNBC).

**Methods**: Using a panel of pre-clinical TNBC cell lines, we measured the sensitivity to prexasertib. We examined the effect activation of EGFR had on prexasertib sensitivity. We measured the synergy of dual blockade of EGFR with erlotinib and CHK1 with prexasertib in TNBC cell lines and xenografts.

**Results**: EGFR overexpression and activation increased resistance to CHK1 inhibition by prexasertib. EGFR promoted the phosphorylation of BCL2-associated agonist of cell death (BAD), inactivating its pro-apoptotic functions. Inhibition of EGFR reversed BAD phosphorylation, increasing sensitivity to prexasertib.

**Conclusion**: The use of prexasertib as a monotherapy in TNBC has been limited due to modest clinical responses. We demonstrated that EGFR activation contributes to innate resistance to prexasertib in TNBC and potentially other cancers. EGFR expression status should be considered in clinical trials examining prexasertib’s use as a monotherapy or combination therapy.

## Introduction

Triple negative breast cancer (TNBC) is an aggressive form of breast cancer associated with poor prognosis and metastasis. TNBC is characterized by the lack of estrogen receptor (ER^-^), progesterone receptor (PR^-^), and the growth factor receptor HER2/erbB2 (HER2^-^). The lack of these targetable molecules for hormone and growth factor therapy limit current treatment options for TNBC.

Given PARP inhibitors’ success in BRCA mutated breast cancers, there has been a renewed focus on molecular inhibitors for DNA damage and response (DDR) proteins, such as ATM, ATR, and CHK1, as molecular targets in aggressive cancers such as TNBC^[1]^. Inhibition of these critical cell cycle regulators enhance replication stress in proliferating cancer cells and promote synthetic lethality with defects in homologous recombination^[1,2]^.

Interestingly, activation of ATR/CHK1 has been observed as a method of stabilizing the replication fork and promoting resistance to PARP inhibitors^[3-5]^. CHK1 plays a critical role in response to DNA damage and is an essential effector in the regulation of replication. Inhibitors of CHK1 alter DNA damage response and abrogate S and G_2_-M cell cycle checkpoints to promote replication stress, induce double strand breaks, and promote cell death through mitotic catastrophe in highly proliferating cells^[6]^. In the last several years, CHK1 inhibitors, such as prexasertib (LY2606368), have shown success in promoting cell death as a monotherapy in high grade serous ovarian cancer, squamous cell carcinoma, and neuroblastoma^[5,7-9]^.

The lack of molecular targets in TNBC has prompted investigations into CHK1 inhibitors as single-agent or combination therapies^[10]^. Several reports have demonstrated that prexasertib is effective, particularly as combination therapies in TNBC^[11-13]^. However, as a monotherapy, prexasertib has only shown modest activity in the treatment of TNBC (NCT02203513)^[14]^. Modest clinical activity may be due to innate or acquired resistance to prexasertib, which is poorly understood.

In 2019, Lowery *et al.*^[8]^ described innate and acquired resistance to prexasertib in sarcoma xenografts. Prexasertib-resistant tumors showed higher levels of the anti-apoptotic protein BCL-xL and increased phosphorylation along the PI3K and MAPK signaling pathways. Specifically, highly activated AKT, MEK1/2, and ERK1/2 were observed in resistant tumors^[8]^. Increased RAS/MEK/ERK activity has also been reported in response to CHK1 inhibition in other cell lines^[15,16]^. However, combinations of MAPK or PI3K inhibitors with prexasertib were insufficient to overcome acquired resistance in sarcoma xenografts^[8]^.

Innate or acquired resistance to prexasertib may arise from mechanisms that drive cell proliferation and stabilize the replication fork. An upstream regulator of both MAPK and PI3K pathways is the epidermal growth factor receptor (EGFR). EGFR overexpression or activation stimulates RAS/MAPK and, to a lesser extent, PI3K/AKT/mTOR signaling, driving cell proliferation and potentially bypassing prexasertib-induced replication stress. EGFR overexpression is observed in ~50% of TNBC tumors and is associated with poor overall survival^[17-19]^. Therefore, we examined EGFR expression and inhibition in TNBC preclinical cell lines to determine if EGFR promoted resistance to prexasertib and if inhibition of EGFR could enhance the anti-tumor activity of prexasertib in TNBC tumors.

## Methods

### Cell culture

The TNBC cell lines HCC1806, HCC1937, MDA-MB-157 (MDA-157), MDA-MB-231 (MDA-231), and MDA-MB-468 (MDA-468) were purchased from the American Type Culture Collection (Manassas, VA; CRL-2335, CRL-2336, HTB-24, HTB-26, and HTB-132). MX-1 was purchased from the NCI repository. All cell lines were purchased within the previous 24 months and passaged < 15 times for all experiments [Table 1]. Cells were tested biweekly for mycoplasma contamination (MycoAlert, Lonza, Basel, Switzerland). MX-1, MDA-157, MDA-231, and MDA-468 were grown in Dulbecco Modified Eagle Medium (DMEM High Glucose with GlutaMAX, Life Technologies, Carlsbad, CA) and supplemented with 1% sodium pyruvate (Life Technologies) and 10% fetal bovine serum (FBS) (Premium Select, Atlantic Biologicals, Miami, FL). HCC1937 and HCC1806 were grown in RPMI supplemented with 10% FBS. Cells were maintained in a humidified 37 °C incubator with 5% carbon dioxide.

**Table 1:** Mutational status and TNBC subgroup of the cells line used in this work

Cell line	Mutations	Subgroup
HCC1806	TCF12-A482V	Basal A
MDA-MB-157	FAT4-L4468P; MSH6-R644S	Basal B
MDA-MB-231	BRAF-G4646V; CD79A-C106Y; KRAS-G13D; NF2-E231*; PBRM1-I228V; PDGFRA-Y172F; TP53-R280K	Basal B
MDA-MB-468	CACNA1D-E953D; TP53-R273H, PTEN V85_splice	Basal A
HCC1937	TP53-R308; PTEN deletion; BRCA-5396insC	Basal A
MX-1	BRCA1-3363delGAAA	Basal B

TNBC: triple negative breast cancer

### Cytotoxicity

Cytotoxicity of monolayer and 3D cultures were determined with a cell viability assay, CellTiter-Glo and CellTiter-Glo 3D (Promega, Madison, WI). For monolayer experiments, HCC1806, HCC1937, MDA-157, MDA-468, and MX-1 were plated at 5000 cells per well in clear-bottomed white 96-well plates. MDA-231 cells were plated at 2500 cells per well in clear-bottomed white 96-well plates. The cells were allowed to attach for two days, and then they were treated with increasing concentrations of prexasertib (LY2606368, Selleck Chemicals, Houston, TX), erlotinib (Selleck Chemicals), or both agents. The cells were exposed continuously for four days, and the viability was assessed with CellTiter-Glo. For EGF stimulation, MDA-231 or MDA-468 were treated with EGF (human recombinant proteins, Life Technologies, Carlsbad, CA) at 50 or 500 nmol/L at the same time as the indicated concentrations of prexasertib were added. Cells were exposed continuously for four days and viability assessed with CellTiter-Glo.

For spheroid growth, clear-bottomed white 96-well plates were coated with 50 µL of Corning Matrigel Growth Factor Reduced (GFR) Basement Membrane Matrix (Fisher Scientific, Waltham, MA). MDA-231 and MDA-468 cells were then seeded at 5000 cells per well on top of the gel layer in DMEM with 2.5% Matrigel. The cells were grown for four days to allow spheroids to form. The cells were then treated with increasing concentrations of prexasertib, erlotinib, or both agents. Cells were exposed continuously for four days, and the viability was assessed with CellTiter-Glo 3D.

The assay reagent (CellTiter-Glo or CellTiter-Glo 3D) was added to plates and incubated according to the manufacturer’s instructions. Luminescence was read on a multimodal plate reader (Infinite M1000, Tecan, Männedorf, Switzerland). All viability assays were performed with technical triplicates over three biological replicates. The results were normalized to values for cells exposed to vehicle control and graphed to generate half-maximal inhibitory concentration (IC_50_) using software (Prism, GraphPad, San Diego, CA).

### Combination index analysis

Combination Index (CI) values were analyzed using the readily available CompuSyn software^[20]^. Percent survival data of 3D combination experiments were entered as a decimal where 1.00 is equal to 100% survival and 0.00 is equal to 0% survival. Single compound data, as well as combination data, were entered and the non-constant drug combination analysis was utilized to generate CI values where a value > 1 represents an antagonist effect, a value of < 1 represents a synergistic effect, and a value of 1 represents an additive effect^[20]^.

### Immunoblot

Immunoblotting was performed as described previously^[21]^. Cells were grown in 10-cm dishes and cultured to 70%-80% confluence. Cells were rinsed with phosphate-buffered saline, scraped, stored overnight at -80 °C, and lysed. Lysates were separated on a 4%-15% sodium dodecyl sulfate-polyacrylamide gel electrophoresis gel (Bio-Rad, Hercules, CA) and transferred to a nitrocellulose membrane. The membrane was probed with antibodies diluted in 5% nonfat dry milk in Tris-buffered saline (VWR, Radnor, PA) and 0.1% Tween 20 (Fisher Scientific) and raised against reagents for immunoblot [Table 2]. Antibodies were incubated at 4 °C overnight on a rocker. The blots were washed and incubated with horseradish peroxidase (HRP)-labeled secondary antibodies (goat anti-rabbit-HRP or goat anti-mouse-HRP) (Cell Signaling Technology) and diluted 1:5000 for 1 h at room temperature (~23 °C) on a rocker. HRP antibody target proteins were detected by incubating with an HRP substrate (WesternBright Sirius, Advansta, San Jose, CA). All immunoblots were performed in two or more biological replicates.

**Table 2:** Antibodies used in this work

Dilution	Reagent	Source
1:500	p-EGFR Tyr 1068	Cell Signaling, Danvers, MA
1:1,000	AKT BAD BRAF EGFR ERK GAPDH MEK p-AKT Ser 473 p-BAD Ser 112 p-BRAF Ser 445 p-ERK Thr 202/204 p-MEK Ser 217/221	Cell Signaling Cell Signaling Cell Signaling Cell Signaling Cell Signaling Santa Cruz Biotechnology, Dallas, TX Cell Signaling Cell Signaling Cell Signaling Cell Signaling Cell Signaling Cell Signaling
1:30,000	β-actin	Life Technologies

AKT: protein kinase B; EGFR: epidermal growth factor receptor; ERK: extracellular signal-regulated kinase

### Animal care and welfare

All procedures were performed in accordance with guidelines approved by the University of South Alabama’s Institutional Animal Care and Use Committee (IACUC). All animals were allowed access to food and water ad libitum and received veterinary care.

Forty athymic nude mice (Charles River) were implanted subcutaneously with 10^6^ MDA-231 cells or 10^7^ MDA-468 cells 1:1 in low growth factor Matrigel. Nineteen days after MDA-231 tumor implantation and twenty-six days after MDA-468 tumor implantation, mice were dosed with the vehicle, prexasertib (dissolved in 40% captisol and sterile water) alone, erlotinib (dissolved in 4% captisol and sterile water) alone, or a combination of prexasertib and erlotinib. Prexasertib was administered via subcutaneous injection [10 mg/kg body weight (BW)] twice daily for three days, followed by a four-day rest. Erlotinib was administered via oral gavage (50 mg/kg BW) daily. Dosing amounts and schedules were selected based on experiments by Lowery *et al.*^[8]^ looking at combination exposures with prexasertib and other small molecule inhibitors and reports examining erlotinib response of TNBC xenografts^[8,9,22,23]^. Animals were treated for four weeks. Tumor volumes were calculated using the formula V = (L × W × W)/2, where V is volume, L is length, and W is width. Body weights were analyzed twice weekly, and animals were observed for signs of distress or pain and sacrificed when appropriate.

### Statistical analysis

Mean IC_50_ values ± standard error of the mean (SEM) were determined from at least three biological replicates. Tumor volumes, tumor weights, and body weights are presented as mean ± SEM. All values were evaluated with one-way analysis of variance (ANOVA), and means were compared with Dunnett’s *post hoc* test or Tukey’s *post hoc* test. Statistical significance was defined by *P < 0.05, **P < 0.01, and ***P < 0.001.

## Results

### Sensitivity of TNBC cell lines to prexasertib

**Figure 1:**
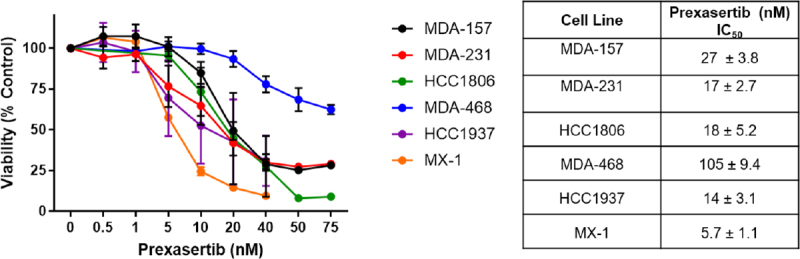
Sensitivity of TNBC cell lines to CHK1 inhibitor prexasertib. IC_50_ values are expressed as mean ± standard error of the mean (SEM). TNBC: triple negative breast cancer

We first examined the sensitivity of a panel of TNBC cell lines to prexasertib [Figure 1]. Prexasertib effectively induced cell death at nanomolar concentrations for most TNBC cell lines, including BRCA mutated HCC1937 and MX-1 [Figure 1 and Table 1]. The highest sensitivity to prexasertib was observed in MX-1 cells (orange line, IC_50_, 5.7 nmol/L), while MDA-468 cells were highly resistant (blue line, IC_50_, 105 nmol/L).

**Figure 2:**
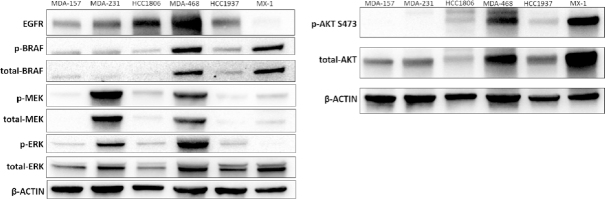
Immunoblot of BRAF/MEK/ERF signaling axis and activated AKT in the TNBC cell line panel. TNBC: triple negative breast cancer; AKT: protein kinase B

Immunoblot analysis of the BRAF/MEK/ERK signaling pathway in the TNBC cell lines showed mixed activation of this pathway in the prexasertib-sensitive cell lines [Figure 2]. In contrast, MDA-468 showed high activation of this pathway, similar to the prexasertib-resistant rhabdomyosarcoma xenograft^[8]^. MDA-468 also contained the highest expression level of EGFR. We examined AKT activation in the TNBC cell line panel and observed high activation of AKT in the MDA-468 and MX-1 cell lines. The activation of other DDR proteins in these cell lines is shown in Supplementary Figure 1.

### EGF stimulation increased resistance to prexasertib in MDA-231 cells

**Figure 3:**
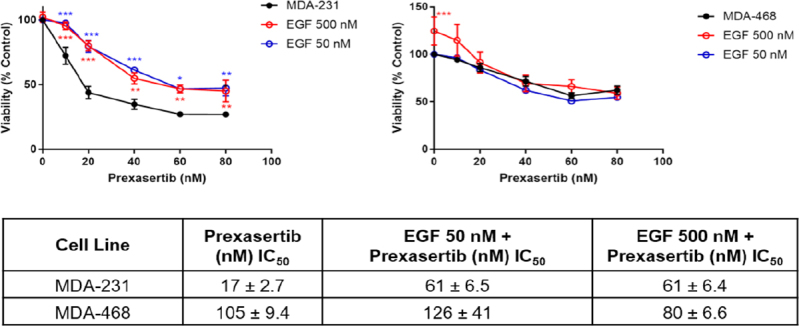
EGF stimulation increased resistance to prexasertib when EGFR is not overexpressed. IC_50_ values are expressed as mean ± SEM. EGF: epidermal growth factor; EGFR: epidermal growth factor receptor; SEM: standard error of the mean

Given the overexpression of EGFR in the MDA-468 cell line and the BRAF/MEK/ERK pathway stimulation, we examined whether stimulation by EGF would reduce the sensitivity of MDA-231 to prexasertib [Figure 3]. The MDA-231 cell line was selected because it has intermediate EGFR expression and sensitivity to prexasertib [Figures 1 and 2]. Stimulation of the MDA-231 cells with both 50 and 500 nmol/L EGF increased resistance to prexasertib with the IC_50_ shifting from 17 to 61 nmol/L [Figure 3]. No change was observed when MDA-468 was stimulated with 50 nmol/L EGF, although a significant increase in cell viability was observed with 500 nmol/L EGF alone.

### Erlotinib increased the sensitivity of MDA-468 to prexasertib

**Figure 4:**
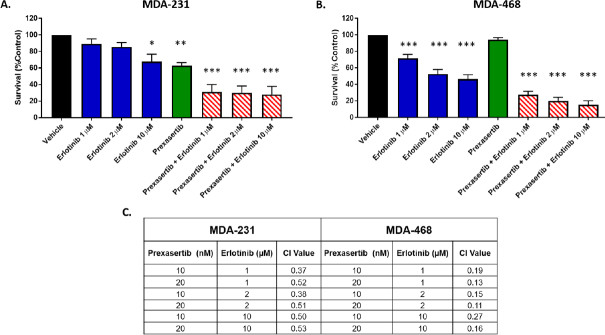
Erlotinib co-exposure with prexasertib synergistically enhanced cell killing. Viability of 3D cultures of MDA-231 (A) and MDA-468 (B) after exposure to prexasertib, erlotinib, and combinations of erlotinib and prexasertib; the combination index (CI) showed synergistic interaction (CI < 1) of erlotinib and prexasertib in both MDA-231 and MDA-468 (C)

With EGF stimulating prexasertib resistance in the MDA-231 cells, we tested the synergy of EGFR inhibition with prexasertib. Given that efficacy can differ between monolayer culture and three-dimensional (3D) culture, we tested the combination of erlotinib and prexasertib in spheroids of MDA-231 and MDA-468 [Figure 4]. MDA-231 showed more resistance to 20 nM prexasertib in 3D culture (viability 63% ± 4.0%) than was observed in monolayer culture (viability 43% ± 4.8%). MDA-468 cells were similarly resistant to prexasertib in monolayer and 3D cultures. MDA-231 were only partially sensitive to erlotinib alone with viabilities of 89 ± 6%, 86 ± 5%, and 68 ± 9% for 1, 2, and 10 µmol/L, respectively [Figure 4A]. MDA-468 showed greater sensitivity to erlotinib with viabilities of 72% ± 5% (*P* < 0.001 compared to control), 52 ± 5% (*P* < 0.001 compared to control), and 47% ± 5% (*P* < 0.001 compared to control) for 1, 2, and 10 µmol/L, respectively [Figure 4B]. Combining prexasertib with erlotinib showed synergistic interactions for both cell lines with the most pronounced effect seen in the MDA-468 spheroids [Figure 4B and C].

**Figure 5:**
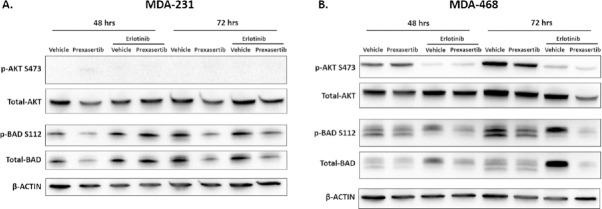
Erlotinib co-exposure with prexasertib reduced BAD phosphorylation. MDA-231 cells treated with vehicle, 20 nmol/L prexasertib, 10 µmol/L erlotinib, or both 20 nmol/L prexasertib and 10 µmol/L erlotinib at 48 and 72 h (A); MDA-468 cells treated with vehicle, 20 nmol/L prexasertib, 10 µmol/L erlotinib, or both 20 nmol/L prexasertib and 10 µmol/L erlotinib at 48 and 72 h (B)

Immunoblotting of MDA-231 and MDA-468 treated with prexasertib, erlotinib, and both prexasertib and erlotinib showed that the co-exposures reduced the phosphorylation of BCL2-associated agonist of cell death (BAD), releasing the pro-apoptotic protein from 14-3-3 sequestration [Figure 5]^[24]^. A reduction in activated AKT was also observed in the prexasertib-resistant MDA-468.

**Figure 6:**
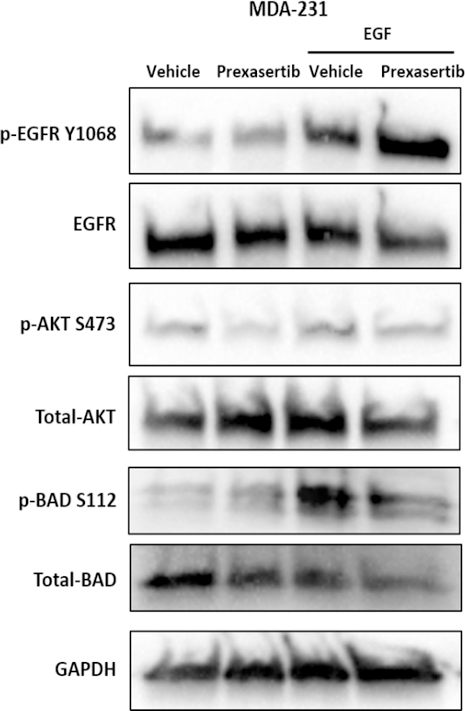
EGF stimulation in MDA-231 increased BAD phosphorylation. MDA-231 cells treated with vehicle, 20 nmol/L prexasertib, 50 nmol/L EGF, or both 50 nmol/L EGF and 20 nmol/L prexasertib were examined after 48 h of exposure. AKT: protein kinase B; EGF: epidermal growth factor; EGFR: epidermal growth factor receptor

Immunoblotting of EGF stimulated MDA-231 cells also confirmed that EGF activation increased phosphorylation of BAD at serine 112 (S112), reducing apoptotic activity and promoting resistance to prexasertib [Figure 6].

### Co-exposure of erlotinib and prexasertib promoted tumor reduction in prexasertib-resistant MDA-468 xenografts

**Figure 7:**
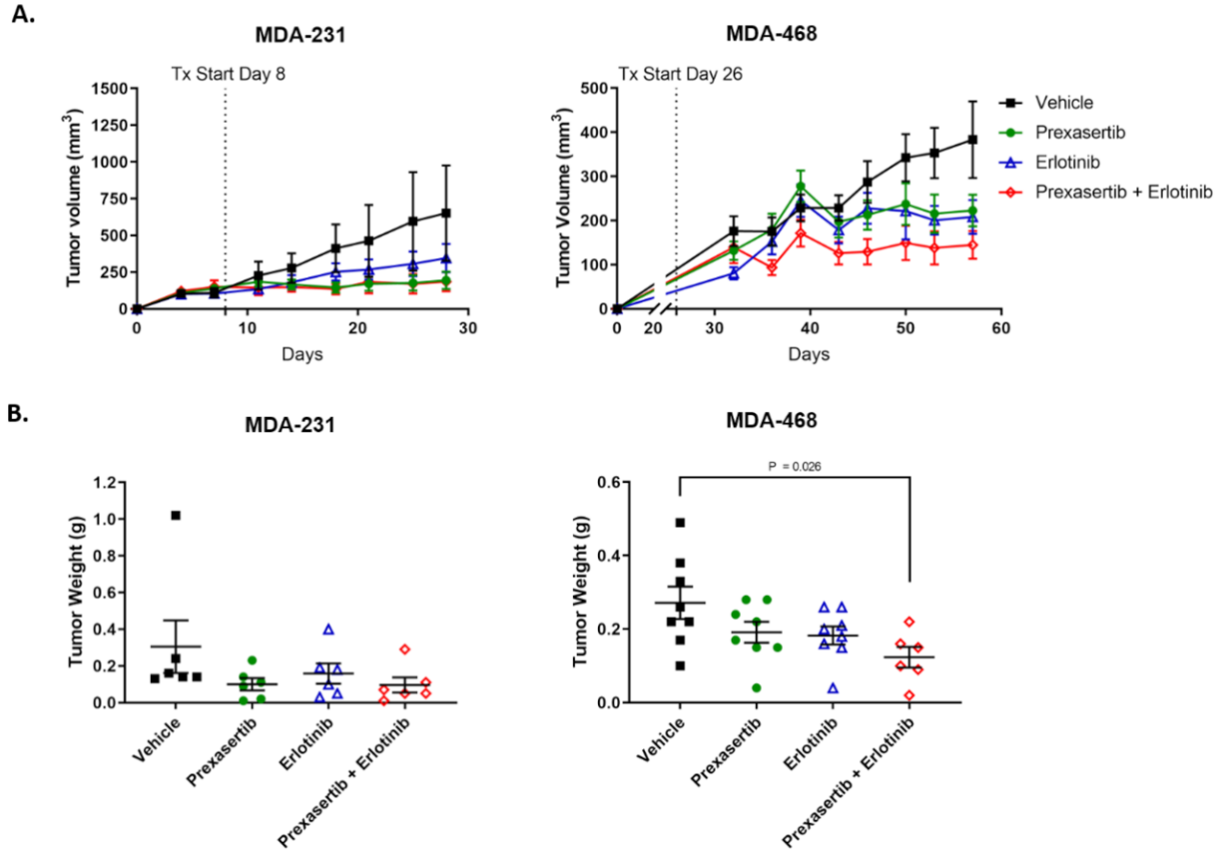
Co-exposure of erlotinib and prexasertib significantly reduced tumor progression in MDA-231 and MDA-468 xenografts. MDA-231 and MDA-468 xenograft containing mice were treated with vehicle, 10 mg/kg BW prexasertib, 50 mg/kg BW erlotinib, or both prexasertib and erlotinib (A); tumor weights at sacrifice for MDA-231 and MDA-468 (B)

Finally, we examined the *in vivo* efficacy of co-dosing erlotinib and prexasertib in MDA-231 and MDA-468. MDA-231 is a commonly used preclinical model cell for TNBC with representative sensitivity to prexasertib [Figure 1]. MDA-468 was selected for its resistance to prexasertib. Athymic nude mice were implanted with MDA-231 or MDA-468. Mice were dosed with vehicle control, prexasertib alone, erlotinib alone, or a combination of prexasertib and erlotinib. MDA-231 tumors showed similar sensitivity to prexasertib, erlotinib, and combination treatment [Figure 7A and B]. MDA-468 tumors showed a significant reduction in tumor volume after combination treatment (from 338 ± 245 mm^3^ to 145 ± 77.3 mm^3^, *P* = 0.026, Figure 7A and B). A non-significant reduction in tumor volume was also observed with prexasertib (208 ± 108 mm^3^) and erlotinib alone (223 ± 101 mm^3^). No significant decrease in body weight was observed during monotherapy or combination therapy in either xenograft model [Supplementary Figure 2].

**Figure 8:**
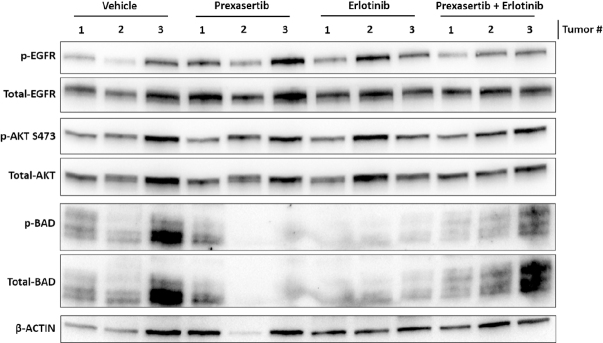
Immunoblotting of MDA-468 xenografts. MDA-468 xenograft tumors were excised and examined for the indicated protein expression. Three tumors per group were analyzed. AKT: protein kinase B; EGFR: epidermal growth factor receptor

Immunoblot of the final MD-468 tumors showed a trend for reduced EGFR activity in tumors treated with both prexasertib and erlotinib [Figure 8]. While the tumors did not show a reduction in AKT phosphorylation in any of the groups, there was an indication of a reduction in BAD phosphorylation consistent with in vitro work [Figure 5]. Despite the lack of effect on AKT activity, there was still a clear reduction of tumor volume and tumor weight [Figure 7].

## Discussion

Molecular targeting has significantly enhanced survival for hormone and growth factor positive breast cancers. TNBC has remained challenging to treat because effective molecular targets have not been identified. CHK1 inhibitors such as prexasertib have shown efficacy as a monotherapy in difficult to treat tumor types such as neuroblastoma and high-grade serous ovarian tumors^[5,7,9]^. CHK1 inhibitors’ efficacy as monotherapy in TNBC has been limited by modest clinical responses^[14]^. Recent studies have described innate and acquired resistance to prexasertib that could help develop biomarkers to stratify patients for prexasertib monotherapy treatment.

When characterizing prexasertib in 2015, King *et al*.^[6]^ noted that the colorectal cancer cell line HCT116 and pancreatic carcinoma PANC-1 showed no sensitivity to prexasertib. In 2019, Lowery *et al.*^[8]^ demonstrated the acquired resistance to prexasertib in a rhabdomyosarcoma xenograft and innate resistance in Ewing’s sarcoma and osteosarcoma cell lines. Acquired resistance was characterized by the activation of the PI3K and MAPK pathways, but there was no characterization of the pathways involved in innate resistance. More recently, Nair *et al.*^[25]^ observed acquired resistance in BRCA wild-type high-grade serous ovarian cancers and attributed resistance to downregulation of CDK1/cyclin B1 prolonging G2 and delaying mitotic catastrophe. Contributions of the PI3K and MAPK pathways were not examined.

While acquired resistance may contribute to the modest clinical response observed with prexasertib in TNBC, innate resistance in TNBC tumors may also be a driving factor. We currently lack biomarkers for intrinsic resistance to prexasertib in TNBC and other cancers. Examining a panel of *BRCA* wild-type and *BRCA* mutated TNBC cell lines, we noted innate resistance to prexasertib in the MDA-468 cell line [Figure 1]. Immunoblotting showed highly activated MAPK pathways in this cell line, similar to the results reported by Lowery *et al.*^[8]^ for acquired resistance [Figure 2]. We also observed AKT activation, but both the MDA-468 and MX-1 showed highly activated AKT, and they correspond with the most and least resistance to prexasertib, respectively [Figures 1 and 2]. Critically, we also noted overexpression of EGFR in the MDA-468 cell line, which is an upstream effector of the MAPK pathway and, to a lesser extent, the PI3K pathway. Given that Lowery *et al.*^[8]^ observed no reversal of prexasertib resistance with MAPK inhibitors, we examined EGFR’s role in innate prexasertib resistance.

Stimulation of EGFR by EGF in the MDA-231 cells resulted in increased resistance to prexasertib [Figure 3]. Inhibition of EGFR with erlotinib showed synergistic activity with prexasertib in MDA-231 and MDA-468 [Figure 4]. Immunoblotting of combination therapy showed that phosphorylation of BAD was downregulated after erlotinib treatment, promoting the release of BAD from 14-3-3 and signaling of apoptosis^[24]^ [Figure 5]. The importance of phosphorylated BAD was further confirmed in EGF stimulated MDA-231 cells, where EGF increased phosphorylated BAD promoting resistance to prexasertib [Figure 6].

Tumor xenografts of MDA-468 confirm the synergistic activity of prexasertib and erlotinib *in vivo*, although there was no apparent benefit to combination treatment in MDA-231 xenografts [Figure 7]. While synergy between prexasertib and erlotinib was observed for MDA-231 in 3D culture [Figure 4], the lack of synergy in the *in vivo* experiments is likely due to higher dosing of prexasertib and erlotinib in the *in vivo* experiments. Erlotinib dosing for the xenograft models was selected based on existing literature and known sensitivity of TNBC cell lines^[22,23]^. Prexasertib dosing was also selected based on existing literature^[8,9]^. The synergy of the 3D experiments and the high tumor reduction observed in our animal experiments suggest future dosing experiments should be conducted to optimize the prexasertib to erlotinib ratios to minimize side effects, while maintaining tumor reduction. Myelosuppression is commonly a dose-limiting factor for CHK1 inhibitors in the clinical setting^[1]^. We did not see significant toxicity or weight loss with prexasertib, erlotinib, or combination treatment in either xenograft model [Supplementary Figure 2]. However, further dose optimization of the combination treatment could reduce adverse effects while maintaining tumor reduction. Our results are also consistent with those of Zeng *et al.*^[26]^, who reported enhanced cell killing and tumor targeting with cetuximab and prexasertib in head and neck squamous cell carcinoma. Prexasertib resistance was not reported in their xenograft models.

EGFR overexpression has been noted in at least 50% of TNBCs, which is higher than other breast cancer subtypes^[17-19,27]^. Our data indicate that activation or overexpression of EGFR contributes to innate resistance to prexasertib in TNBC and may contribute to the modest clinical efficacy observed in phase I and II trials. Stratification of prexasertib clinical data based on EGFR expression status could offer new insight into the clinical use of prexasertib as a monotherapy for TNBC and other cancers. It should be noted that EGFR overexpression is also observed in HCT116 and PANC-1 cell lines, which have an innate resistance to prexasertib^[6,28,29]^. Ewing’s sarcoma and osteosarcoma also have a significant percentage of EGFR overexpression, which may contribute to their observed innate resistance to prexasertib^[8,16,30,31]^. In 2017, Lee *et al.*^[16]^ noted the complex molecular interactions of the RAS/RAF/MEK/ERK pathways with CHK1 inhibitor sensitivity. The data presented here support EGFR as a biomarker for the use of prexasertib and potentially other CHK1 inhibitors.

## Declarations

### Availability of data and materials

Data and materials described in this manuscript can be made available upon request.

### Copyright

© The Author(s) 2020.
