# Supplementary material for: EGFR signaling promotes resistance to CHK1 inhibitor prexasertib in triple negative breast cancer
Source: Cancer Drug Resist. 2020 Dec 5;3(4):980–91. doi: 10.20517/cdr.2020.73 (PMC8992551; doi:10.20517/cdr.2020.73)
Supplement: Supplementary file 1 [file cdr-3-980-SupplementaryMaterials.pdf]

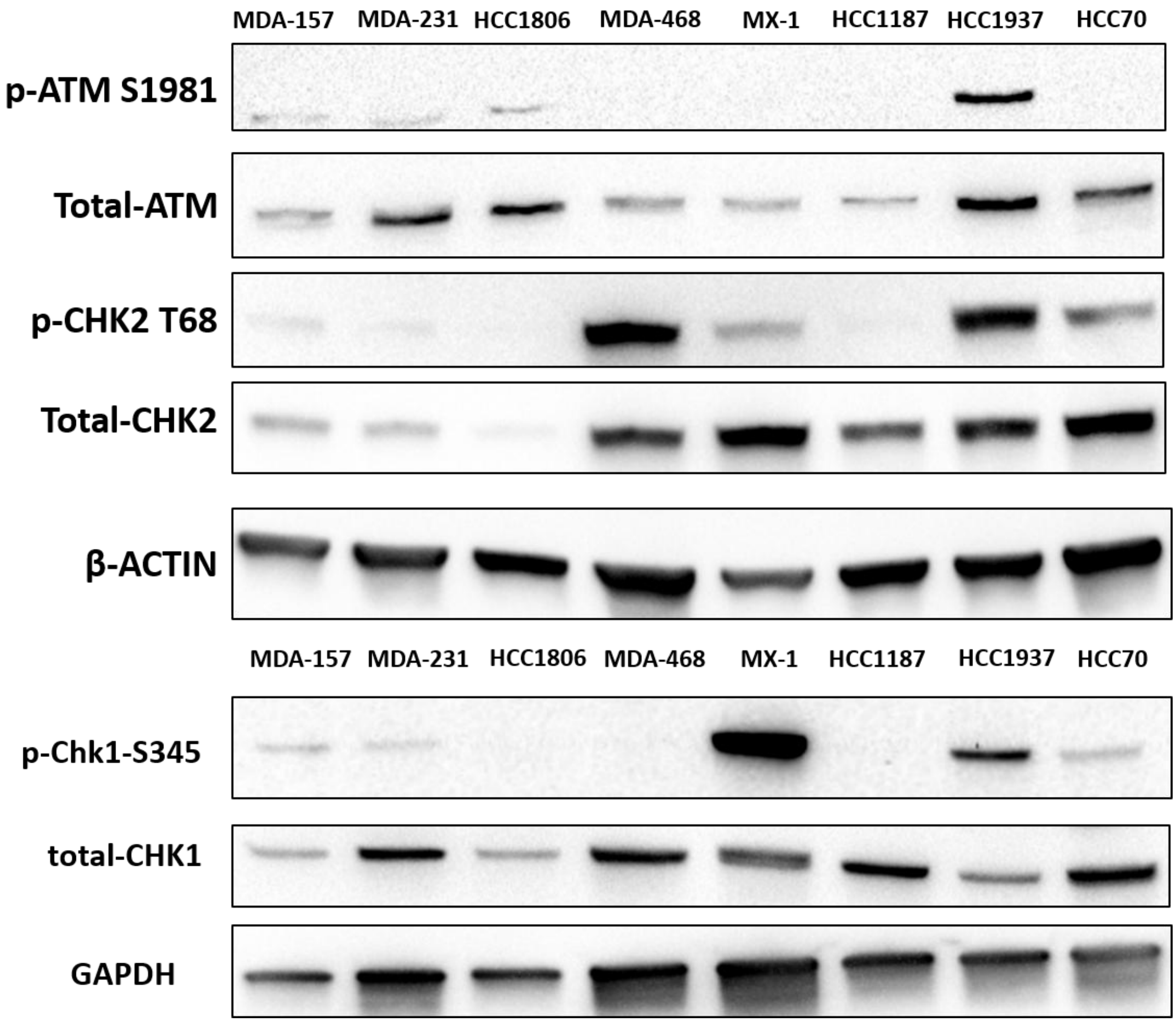

Supplementary Figure 1. Activation of DDR pathway in the TNBC cell panel. DDR: DNA damage and response; TNBC: triple negative breast cancer

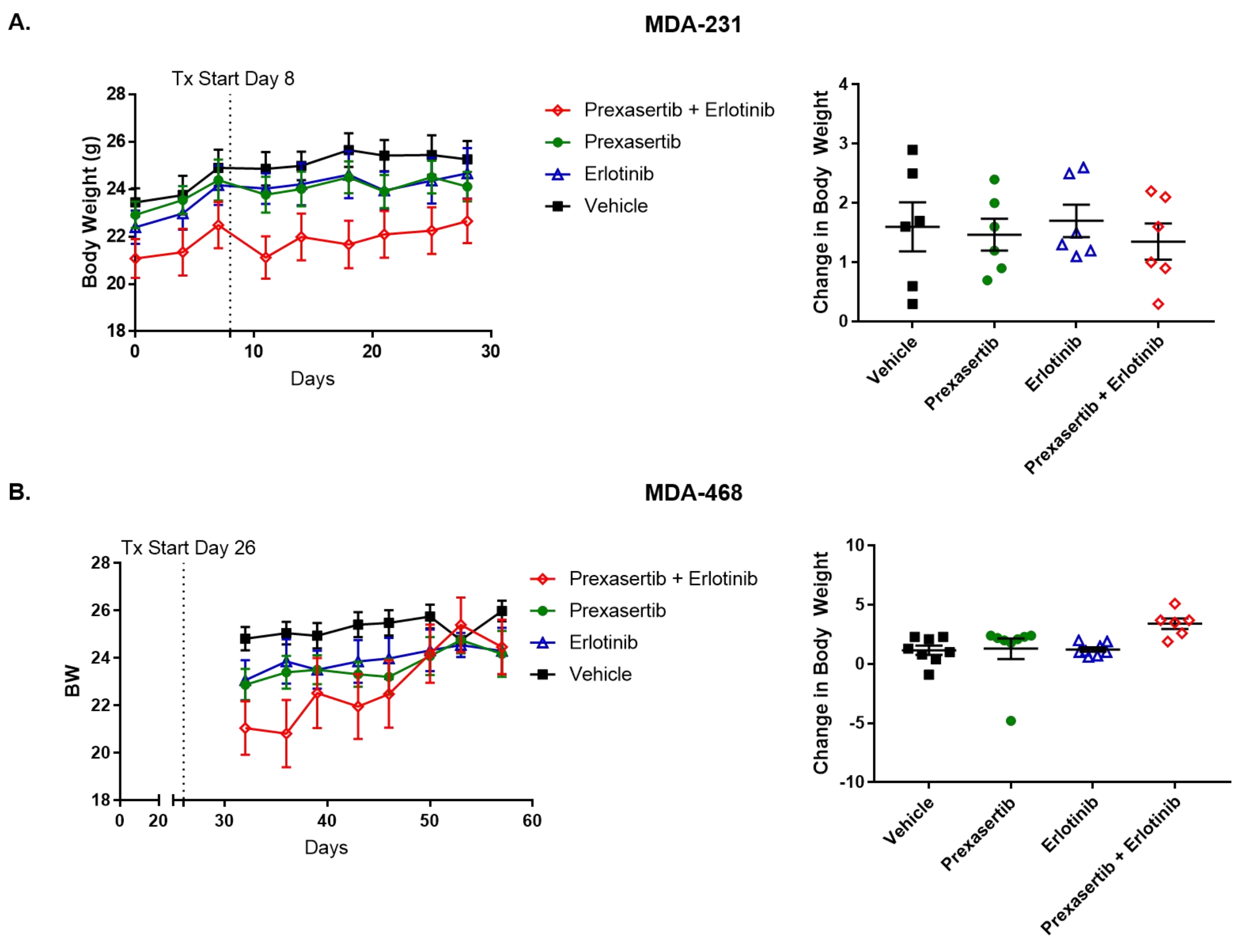

Supplementary Figure 2. Body weight measurements in MDA-231 (A) and MDA-468 (B) xenograft containing mice during treatment with prexasertib, erlotinib, and both prexasertib and erlotinib
